# La sténose urétérale post infectieuse et la mégacalicose: un train qui en cache un autre

**DOI:** 10.11604/pamj.2015.22.334.6868

**Published:** 2015-12-04

**Authors:** Babacar Sine, Ndeye Aissatou Bagayogo, Boubacar Fall, Yaya Sow, Amath Thiam, Alioune Sarr, Abdou Razak Hamidou Zakou, Samba Thiapato Faye, Babacar Diao, Papa Ahmed Fall, Alain khassim Ndoye

**Affiliations:** 1 Service d'Urologie-Andrologie de l'Hôpital Aristide Le Dantec de Dakar, Dakar, Sénégal

**Keywords:** Uropathie, mégacalicose, sténose, infectieuse, uropathy, megacalycosis, stenosis, infectious

## Abstract

La mégacalicose est une uropathie malformative rare caractérisée par une dilatation non obstructive des calices avec hypoplasie de la médullaire rénale. Nous rapportons un cas de mégacalicose associée à une sténose urétérale bilatérale d'origine infectieuse.

## Introduction

La mégacalicose est une uropathie malformative rare caractérisée par une dilatation non obstructive des calices avec hypoplasie de la médullaire rénale. Elle est asymptomatique et n'est découverte qu'au stade de complications. L'association à la sténose urétérale d'origine infectieuse est rare et pose de réels problèmes diagnostiques.

## Patient et observation

**Figure 1:**
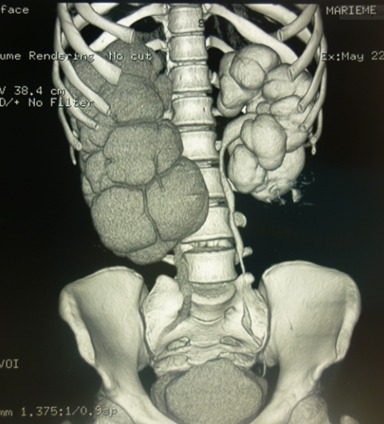
Uro-TDM préopératoire montrant une urétéro-hydronéphrose droite et une dilatation calicielle gauche avec un bassinet de taille normale sans obstacle visualisé

**Figure 2:**
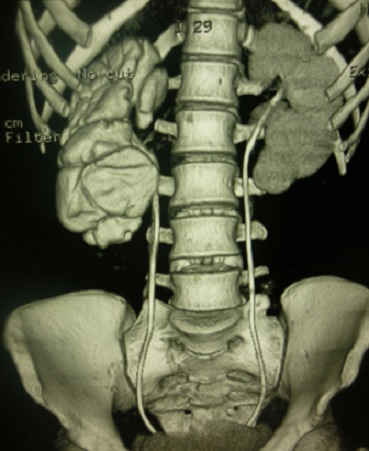
Uro-TDM de contrôle mettant en évidence une persistance de la dilatation des deux reins malgré la présence des sondes JJ

Madame M.B, 19 ans a consulté dans notre service pour des douleurs lombaires droites évoluant depuis un an. Dans ses antécédents, il existait une notion d'hématurie terminale dans l'enfance et une chirurgie abdominale non précisée. L'examen avait mis en évidence une sensibilité des points urétéraux supérieur et moyen droit. Sur le plan biologique, la créatininémie était normale et l'examen cytobactériologique des urines (ECBU) avait isolé un Escherichia coli sensible aux aminosides. L’échographie de l'arbre urinaire avait mis en évidence une urétéro-hydronéphrose bilatérale sans obstacle visualisé. La cystoscopie avait objectivé la présence de granulations réfringentes trigonales. L'uro-tomodensitométrie avait montré (Figure 1): une urétéro-hydronéphrose droite sans obstacle visualisé; une dilatation calicielle gauche avec un bassinet de taille normale sans obstacle visualisé. Ainsi le diagnostic de sténose urétérale droite associée à une mégacalicose gauche avait été retenu et l'indication d'une exploration chirurgicale posée. A l'exploration par laparotomie médiane sous ombilicale, il existait une dilatation urétérale bilatérale en amont d'une sténose de l'uretère lombaire gauche et de l'uretère rétro-méatique droit. Une réimplantation urétéro-vésicale droite selon Cohen a été réalisée et une urétérectomie segmentaire plus anastomose termino-terminale sur sonde JJ bilatérale. Il existait une sténose de l'uretère lombaire droit franchissable par la sonde. Les suites opératoires ont été simples. La patiente a été revue 3 mois après l'intervention, elle n'avait aucune plainte. La créatininémie était normale et l'uro-tomodensitométrie de contrôle (Figure 2) avait mis en évidence une persistance de la dilatation des deux reins malgré la présence des sondes JJ. L'examen anatomo-pathologique de la pièce d'urétérectomie a mis en évidence une inflammation non spécifique.

## Discussion

La mégacalicose est une malformation congénitale du rein décrite en 1963 par Puigvert [1]. Elle est caractérisée par une dilatation non obstructive des calices sans dilatation du bassinet avec hypoplasie de la médullaire rénale. L’éthiopathogénie la plus retenue est l'hypertrophie des calices développée aux dépens d'une hypoplasie primitive de la médullaire rénale [1, 2], le nombre réduit de glomérules juxtamédullaires appuie cette théorie. L'autre théorie est l'existence d'une obstruction in-utero spontanément résolutive avec dilatation séquellaire des calices [2]. Le diagnostic suspecté à l’échographie est confirmé par l'Uro-tomodensitométrie qui montre des calices dilatés contrastant avec un bassinet de volume et de forme normaux et des tiges calicielles et une jonction pyélo-urétérale perméables [2]. La mégacalicose reste longtemps asymptomatique. Son diagnostic ne se fait souvent qu’à l'occasion d'une complication lithiasique ou infectieuse, ou d'une échographie ou uro-TDM réalisée pour une autre affection rénale. Son diagnostic différentiel principal est le syndrome de la jonction pyélo-urétérale qui est rapidement éliminé par l'uro-TDM qui met en évidence souvent un bassinet globuleux avec des calices dilatés en boules. La sténose urétérale est une diminution pathologique permanente et définitive du calibre de la lumière urétérale [3, 4]. Le diagnostic est fait souvent par l'imagerie qui montre l'arrêt de progression du produit de contraste dans l'uretère sur le cliché d'UIV à l'uro-TDM, à l'UPR ou à la pyélographie descendante. De courtes séries d'association de la mégacalicose au mégauretère ont été publiés [5, 6] ainsi que quelques cas cliniques [7, 8]. L'association de la mégacalicose à une sténose de l'uretère d'origine infectieuse est rarissime et n'a jamais été décrit. C'est également une association qui rend difficile le diagnostic de la mégacalicose. En effet en cas d'urétéro-hydronéphrose compliquant la sténose urétérale, il est impossible d’évoquer cette hypothèse. C'est quasiment qu'après avoir traité la sténose de l'uretère et obtenu une dilation persistante du rein que le diagnostic est évoqué.

## Conclusion

La mégacalicose est une affection congénitale rare. L'association d'une sténose urétérale peut cacher son existence. Le cas échéant, seul le traitement de la sténose pourrait faciliter sa surveillance.
